# Choroidal thickness measured with swept source optical coherence tomography in posterior staphyloma strongly correlates with axial length and visual acuity

**DOI:** 10.1186/s40942-019-0166-y

**Published:** 2019-07-09

**Authors:** K. V. Chalam, Kumar Sambhav

**Affiliations:** Department of Ophthalmology, Loma Linda University School of Medicine, Loma Linda, CA USA

**Keywords:** Swept source optical coherence tomography, Staphyloma, Choroidal thickness

## Abstract

**Purpose:**

To measure the choroidal thickness in patients with high myopia from staphyloma using swept source OCT (SS-OCT) in Early Treatment Diabetic Retinopathy Study (ETDRS) fields and compare to normal cohort. The study also evaluated the correlation between choroidal thickness with axial length and best-corrected visual acuity (BCVA).

**Methods:**

In this prospective cross sectional study, 37 eyes of 20 patients with high myopia from staphyloma and 86 eyes of 43 normal subjects were included. In each eye, horizontal scans centered on the fovea (12 × 9 mm) were performed using SS-OCT (DRI-OCT, Topcon, Japan). Choroidal thickness in 9 ETDRS subfields, including central subfield (CSF) was analyzed and correlated with axial length as well as BCVA.

**Results:**

The axial length and BCVA in the high myopia from staphyloma group ranged from 25.12 to 33.54 mm (28.4 ± 1.2) and 20/20–20/400, respectively. The choroidal thickness in staphyloma group was 85.53 ± 48.61 μm as compared to 250.24 ± 71.01 μm in the normal group (*p* < 0.0001). Stepwise regression analysis showed that axial length strongly correlated with decreased choroidal thickness (*p* < 0.001) in staphyloma group (*r* = 0.71, *r*_2_ = 0.5). Refractive error and BCVA moderately correlated(*r* = − 0.47; *r*_2_ = 0.22) with choroidal thickness (*p* < 0.001).

**Conclusion:**

Choroidal thickness in staphyloma (measured with novel technology of SS-OCT) patients is markedly reduced compared to normal controls. In addition, choroidal thickness strongly correlated with axial length of the eye and inversely correlated with visual acuity. This testing modality maybe used as a good predictor of visual acuity in patients with high myopia from staphyloma.

## Introduction

Posterior staphyloma, an abnormal protrusion of the uveal tissue due to weakness of outer layer of eye (sclera) typically involves macula, causes increase in axial length of eye and thereby high myopia. Tessellated fundus, choroidal neovascular membrane (CNV), lacquer cracks, atrophy, stretched vessels, peripapillary atrophic crescent, hemorrhages, tilting of the optic disc often accompany posterior staphyloma [1]. Histologically, the distance between the Bruch’s membrane and the sclera is markedly decreased and thinning of the choroid is noted (30 μm vs. 250 μm in normals) with axial elongation of globe in myopic patients [2, 3]. This thinning of choroid is associated with loss of choriocapillaris and retinal pigment epithelium.

The choroid is known to play a dynamic role in the normal functioning of the eye, and is associated with multiple chorio-retinal diseases such as age-related macular degeneration, diabetic retinopathy, pathologic myopia and others [4]. Choroidal thickness (CT) is a good independent predictor of visual acuity in posterior staphyloma, and inversely correlates with degree of refractive error [5, 6]. Imaging of the choroid has been greatly facilitated with use of optical coherence tomography (OCT). Accurate measurement of choroidal thickness is difficult with spectral domain OCT (SD-OCT) or enhanced depth imaging (EDI) due to limited penetration and hence, poor delineation of the deeper posterior border of the choroid. In contrast, swept-source OCT (SS-OCT) system uses a light source with a long wavelength-sweeping laser (1050 nm) and offers better imaging of the choroid because of their deeper penetration and lower dispersion. In addition, the automated segmentation software of SS-OCT system has superior repeatability than manual measurement techniques (EDI SD-OCT). Choroidal thickness measurements between manual segmentation by Spectralis SD-OCT and automated segmentation by SS-OCT (DRI OCT-1) may differ by ~ 50 μm (the measurements by SD-OCT being higher) [4].

In this study, we measured the thickness of choroid in high myopic individuals with a new SS-OCT system and compared them to age-matched controls. Additionally, we investigated the relationship between choroidal thickness and visual acuity.

## Methods

### Inclusion criteria

This was a cross-sectional observational study of 37 eyes of 20 patients with high myopia from staphyloma (− 9D or above) compared to 86 eyes of 43 normal subjects. The study adhered to the tenets of the declaration of Helisinki. The protocol was approved by the institutional review board. Two groups of patients were studied. In the first group patients with myopia more than − 9D (spherical equivalent refractive error of at least − 9D) associated with staphyloma (all types based on Curtin classification) [14] were included. Staphyloma was confirmed with stereoscopic indirect ophthalmoscopy. In the second group, normal subjects with best-corrected visual acuity (BCVA) of 20/25 or better in each eye with no history or clinical evidence of retinal and choroidal disease were included.

Exclusion criteria for staphyloma/high myopia group included history or clinical evidence of retinal/choroidal disease (other than high myopia), glaucoma, and intraocular pressure of more than 21 mm Hg. Refractive change occurring due to other ocular pathologies (e.g. corneal, lenticular) were excluded. Normal subjects with history or clinical evidence of retinal/choroidal disease, glaucoma, intraocular pressure of more than 21 mm Hg, amblyopia, intraocular surgery or laser therapy, and refractive error beyond ± 5.00D were excluded. Subjects with systemic diseases which might impact choroidal thickness (e.g. hypertension) were also excluded.

### Basic examination

**Fig. 1:**
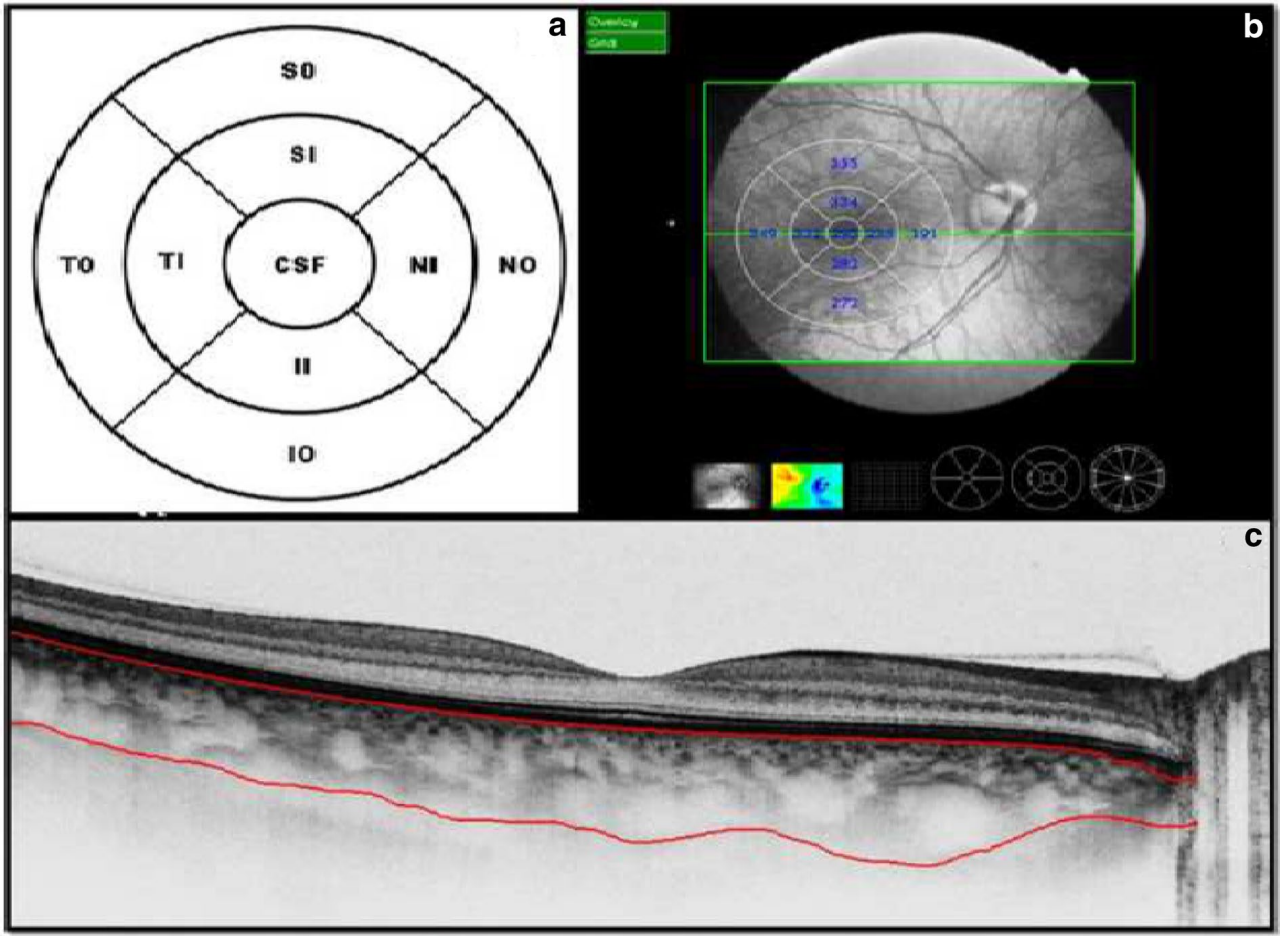
Schematic representation of nine ETDRS segments (**a**) and its overlay over the retinal surface (**b**) along with choroidal thickness for each field. **c** The lines demarcating the choroido-scleral junction and Bruchs choriocapillaris complex

Initial evaluation included BCVA testing (log MAR), applanation tonometry, slit-lamp biomicroscopy, and fundus evaluation. All subjects underwent additional axial length measurement (optical low coherence reflectometry IOL-Master, Carl Zeiss, and Germany). In subjects with poor fixation, axial length was reconfirmed with manual ultrasonography. OCT testing was performed by a single experienced operator using SS-OCT (DRI-OCT 1, Topcon, Tokyo, Japan) with or without pupil dilation and under standardized mesopic light conditions. Data from both eyes of all subjects were used for analyses. Quality of scans was indicated by automated display mode. All the subjects had retinal image quality of ≥ 75% and choroidal image quality of ≥ 80%. The DRI-OCT system uses an axial scan rate of 100,000 Hz using laser wavelength of 1050 nm, yielding an 8-μm axial resolution and transverse resolution of 20μm. All subjects underwent 12 mm × 9 mm radial and 5-line scan centered at the fovea. The choroidal thickness (CT) map contained three concentric rings of diameter 1, 3, and 6 mm with center at fovea and the thickness (the distance between outer choroido-scleral margin and RPE-Bruch’s complex) was obtained automatically with the assistance of SS-OCT software. Inner and outer rings were divided into four segments for a total of nine segments—the central subfield (CSF), superior inner (SI), nasal inner (NI), inferior inner (II), temporal inner (TI), superior outer (SO), nasal outer (NO), inferior outer (IO), and temporal outer (TO) segments (Fig. 1).

### Statistical analysis

The mean choroidal thickness of all nine ETDRS segments were calculated for both the groups and their correlations with axial length and refraction were analyzed in staphyloma group versus control group using the GraphPad Instat 3 (San Diego, California, USA). Snellen visual acuities were converted to logarithm of the minimum angle of resolution (logMAR) for statistical analyses. As a means of exploratory data analysis, bivariate correlations were first performed using Pearson correlation (*r*) among various variables; age, subfoveal choroidal thickness, mean logMAR BCVA and refraction. Correlation between the refractive error (myopic diopter power) and choroidal thickness was also evaluated. *P* value of 0.05 or less was considered statistically significant. Delta (Δ) values (difference of the choroidal thickness from the mean normal values) were calculated and their correlations were drawn with best corrected visual acuity and axial length for staphyloma patients.

## Results

Eighty-six normal eyes from 43 healthy subjects and 37 eyes of 20 staphyloma/high myopia patients underwent clinical examination and SS-OCT evaluation. The ages ranged from 21 to 70 years (median 36 years and mean 40.28 years) in normal subjects and 14–71 years (median 46 years and mean 45.68 years) in staphyloma group. There were 23 women and 20 men included in the normal subject group and 11 women and 9 men in staphyloma group. The distribution according to race is depicted in Table 1. The axial length in staphyloma group ranged from 25.12 to 33.82 mm (28.4 ± 1.2) as compared to 21.79 to 23.89 mm (23.12 ± 0.08) in the normal subjects. In staphyloma group, 22 eyes had a posterior staphyloma (Type 1 and 2 involving macula) whereas 15 eyes had peripapillary staphyloma (Type 3 &4 not involving macula) [14]. BCVA in staphyloma group ranged from 20/20 to 20/400 (logMAR BCVA range 0–1.3). The mean logMAR BCVA in staphyloma group was 0.3 (median 0.4). Four eyes of 2 subjects had mean logMAR BCVA of 0 and two eyes had 1.3. Three eyes in staphyloma group were excluded from analysis (1 patient was mono-ocular and in 2 eyes scan quality did not meet the inclusion criteria). The range of refraction in normal group was + 2.00D to − 5.00D whereas in staphyloma group it was − 9.00D to − 18.00D. All the OCT measurements were done between 8.00 AM to 10.00 AM to reduce the element of diurnal variation in their measurements. Tables 2 and 3 show the breakdown of the mean choroidal thickness in each ETDRS subfield in normal and staphyloma subjects, respectively.

**Table 1:** Comparable baseline demographics between the normal subjects and staphyloma subjects

Parameters	Normal subjects (86 eyes)	Staphyloma subjects (37 eyes)
Age		
Mean	40.28 years	45.68 years
Median	36 years	46 years
Range	21–70 years	14–79 years
LogMAR BCVA	0.0–0.1	0–1.3
Axial length (in mm)	21.79–23.89	25.12–33.82
Sex ratio (male:female)	20:23	9:11
Race	Asian—13	Asian—3
	Caucasian—18	Caucasian—10
	African American—12	African American—7

*BCVA* best corrected visual acuity

**Table 2:** Mean choroidal thickness in nine ETDRS subfield in normal subjects

Parameters	Mean choroidal thickness Mean ± SD (range)			*p* value OD versus OS
	Total cohort	OD (*N* = 43)	OS (*N* = 43)	
CSF	250.24 ± 71.01 (96–401)	252.30 ± 72.10 (96–401)	248.17 ± 69.81 (121–376)	> 0.05
Tl	255.34 ± 66.42 (129–395)	255.65 ± 68.46 (129–395)	255.14 ± 65.13 (137–388)	> 0.05
TO	249.17 ± 58.61 (151–404)	247.93 ± 58.34 (151–396)	250.42 ± 59.53 (155–404)	> 0.05
SI	263.02 ± 69.45 (122–402)	265.32 ± 68.99 (122–396)	260.72 ± 70.7 (122–402)	> 0.05
SO	263.73 ± 65.30 (127–387)	264.77 ± 66.60 (127–387)	262.70 ± 64.8 (146–387)	> 0.05
NI	243.31 ± 71.64 (89–393)	244.74 ± 69.8 (89–383)	241.36 ± 73.6 (94–382)	> 0.05
NO	208.91 ± 70.10 (60–375)	209.44 ± 67.51 (60–358)	206.67 ± 72.51 (78–375)	> 0.05
II	255.34 ± 72.34 (100–438)	259.79 ± 75.06 (100–438)	250.88 ± 69.12 (103–384)	> 0.05
IO	257.98 ± 71.03 (103–443)	260.67 ± 71.89 (121–443)	255.27 ± 70.84 (103–395)	> 0.05

*OD* right eye, *OS* left eye, *CSF* central sub-field, *SI* superior inner, *NI* nasal inner, *II* inferior inner, *TI* temporal inner, *SO* superior outer, *NO* nasal outer, *IO* inferior outer, *TO* temporal outer

**Table 3:** Mean choroidal thickness in nine ETDRS subfield in staphyloma patients

Parameters	Mean choroidal thickness Mean ± SD (range)			*p* value OD versus OS
	Total cohort	OD (*N* = 43)	OS (*N* = 43)	
CSF	78.19 ± 45.07 (11–153)	70.40 ± 39.57 (11–123)	85.53 ± 48.61 (11–153)	> 0.05
TI	88.81 ± 49.36 (22–186)	84.05 ± 43.86 (25–168)	93.31 ± 51.44 (22–186)	> 0.05
TO	96.83 ± 48.44 (26–181)	91.27 ± 43.46 (26–159)	98.10 ± 49.13 (28–181)	> 0.05
SI	88.14 ± 44.13 (20–151)	77.0 ± 39.38 (21–141)	93.16 ± 47.07 (20–151)	> 0.05
SO	87.43 ± 47.74 (13–178)	80.50 ± 44.97 (13–169)	90.00 ± 50.42 (19–178)	> 0.05
NI	75.59 ± 40.66 (19–165)	71.50 ± 40.91 (19–165)	79.47 ± 40.04 (21–162)	> 0.05
NO	57.32 ± 30.22 (13–152)	52.55 ± 29.03 (13–146)	61.84 ± 30.73 (20–152)	> 0.05
II	87.16 ± 44.57 (15–153)	82.22 ± 41.74 (25–142)	91.84 ± 46.60 (15–153)	> 0.05
IO	86.97 ± 44.51 (30–171)	87.00 ± 45.11 (31–171)	86.94 ± 43.94 (30–158)	> 0.05

*OD* right eye, *OS* left eye, *CSF* central sub-field, *SI* superior inner, *NI* nasal inner, *II* inferior inner, *TI* temporal inner, *SO* superior outer, *NO* nasal outer, *IO* inferior outer, *TO* temporal outer

**Fig. 2:**
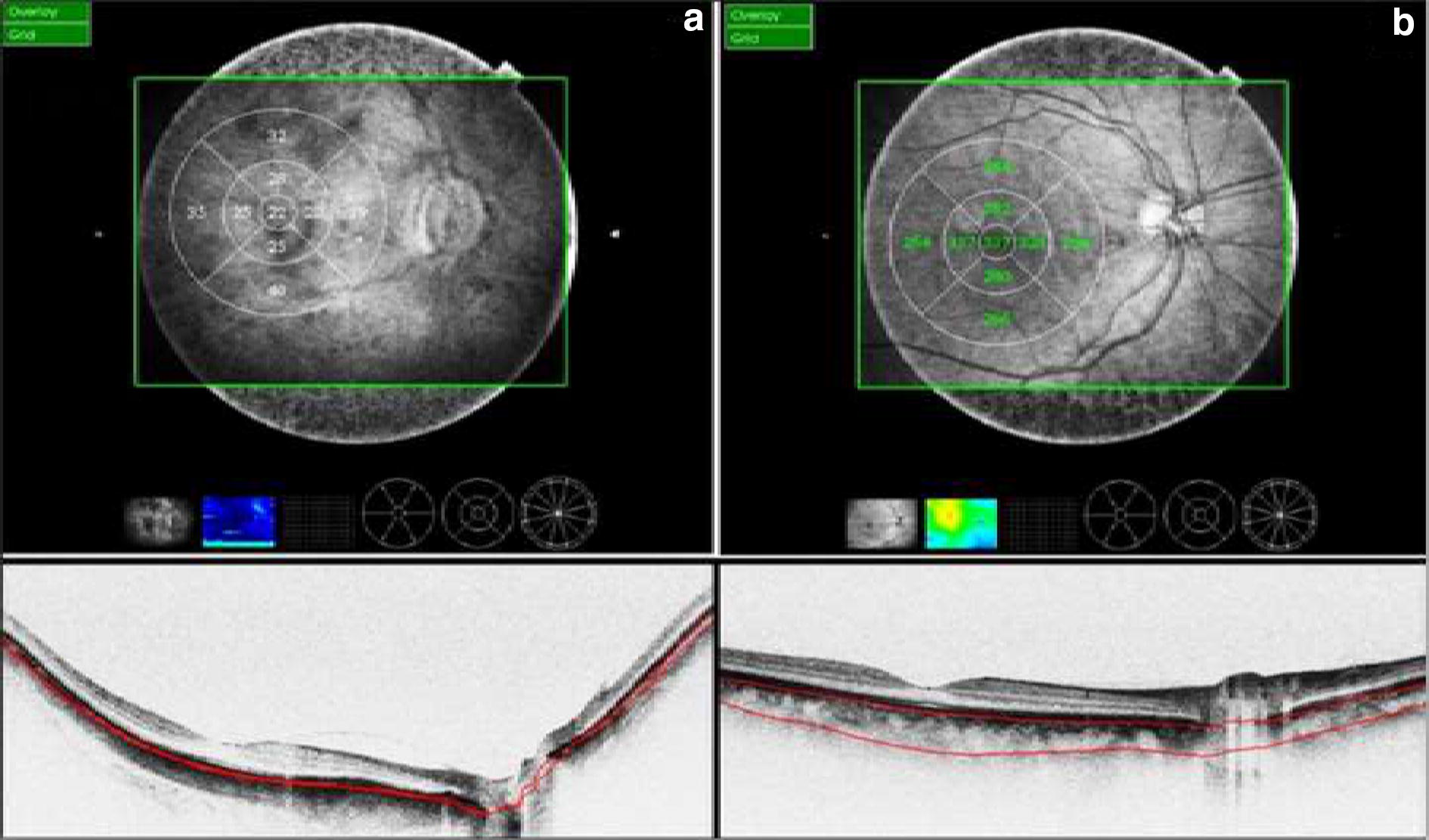
Choroidal thickness in the nine ETDRS segments in patients with staphyloma (**a**) and normal subjects (**b**)

**Fig. 3:**
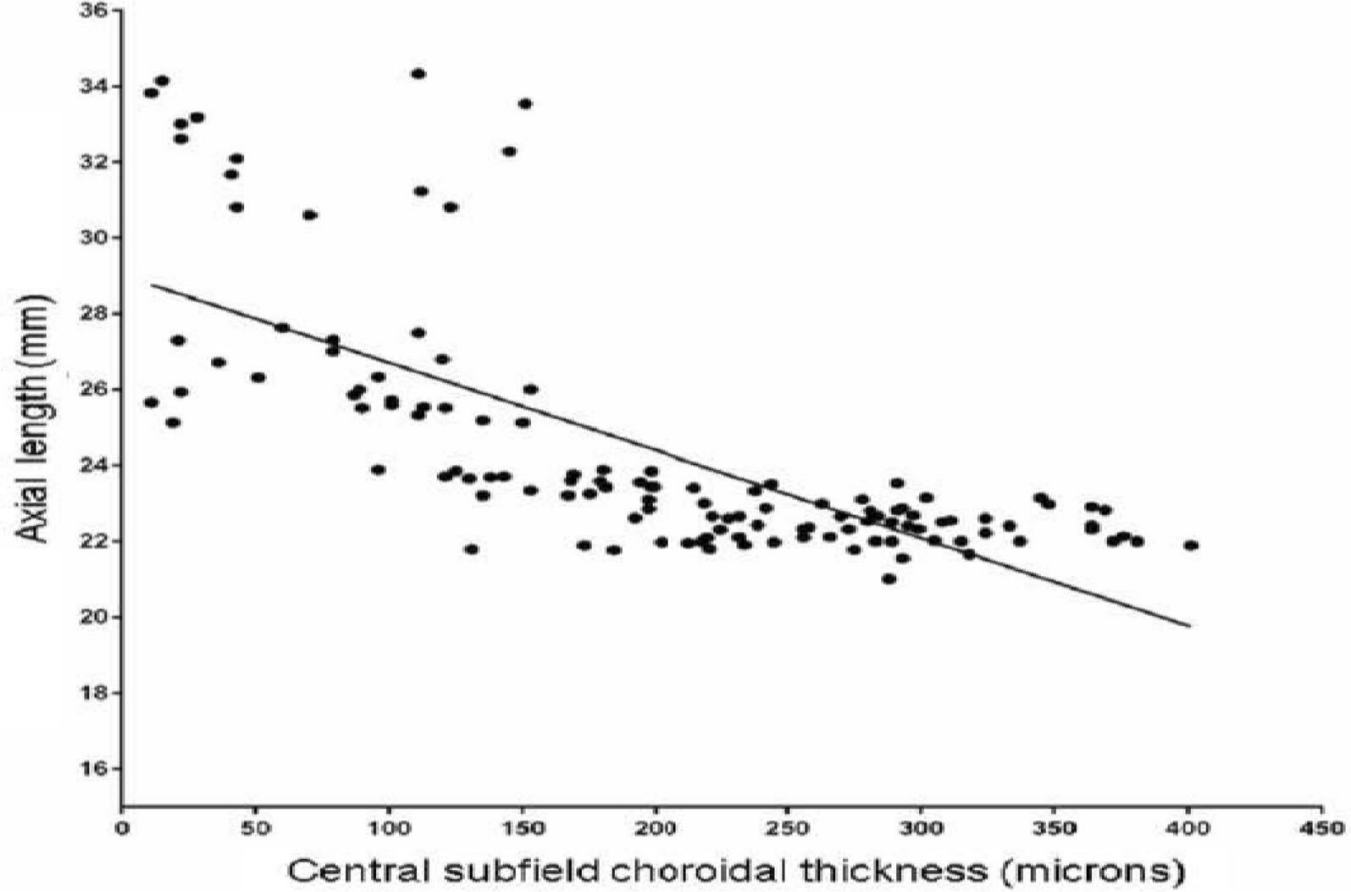
Correlation between central subfield choroidal thickness and axial length in entire cohort (*n* = 123 eyes)

**Fig. 4:**
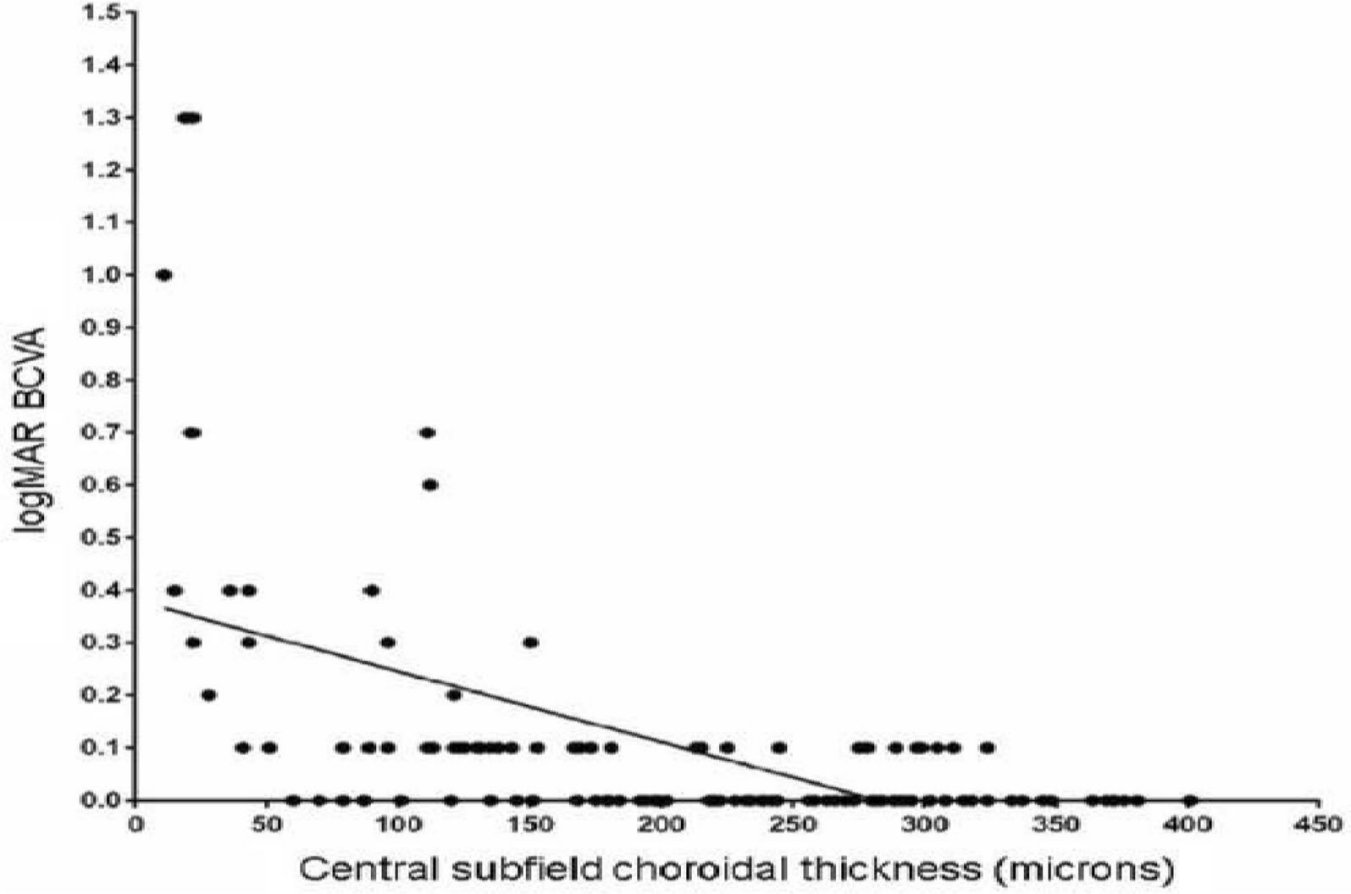
Correlation between central subfield choroidal thickness and log MAR best corrected visual acuity (BCVA) in entire cohort (*n* = 123 eyes)

**Fig. 5:**
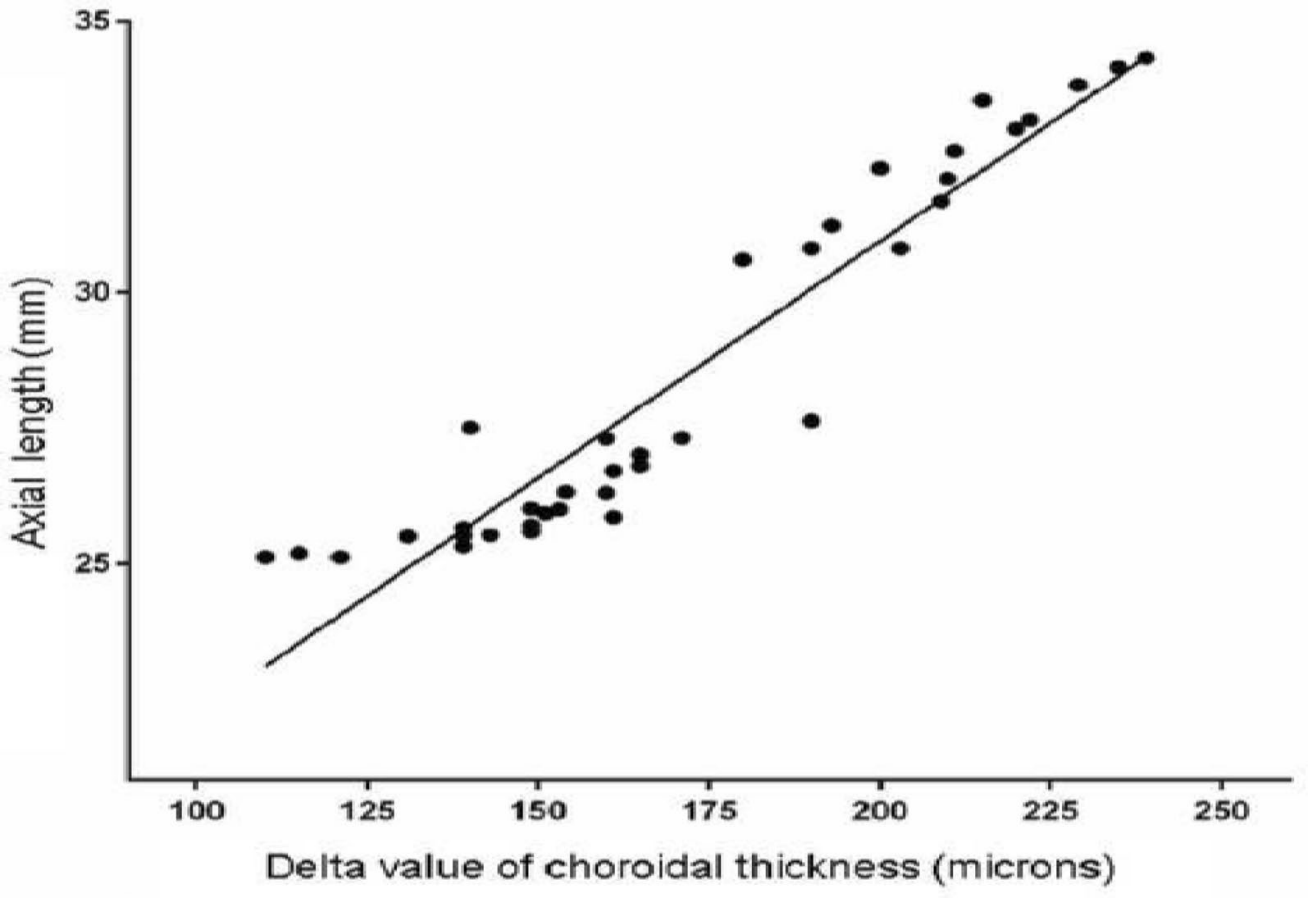
Correlation between delta value (difference between the mean choroidal thickness and actual value) of choroidal thickness and axial length in patients with staphyloma (37 eyes)

**Fig. 6:**
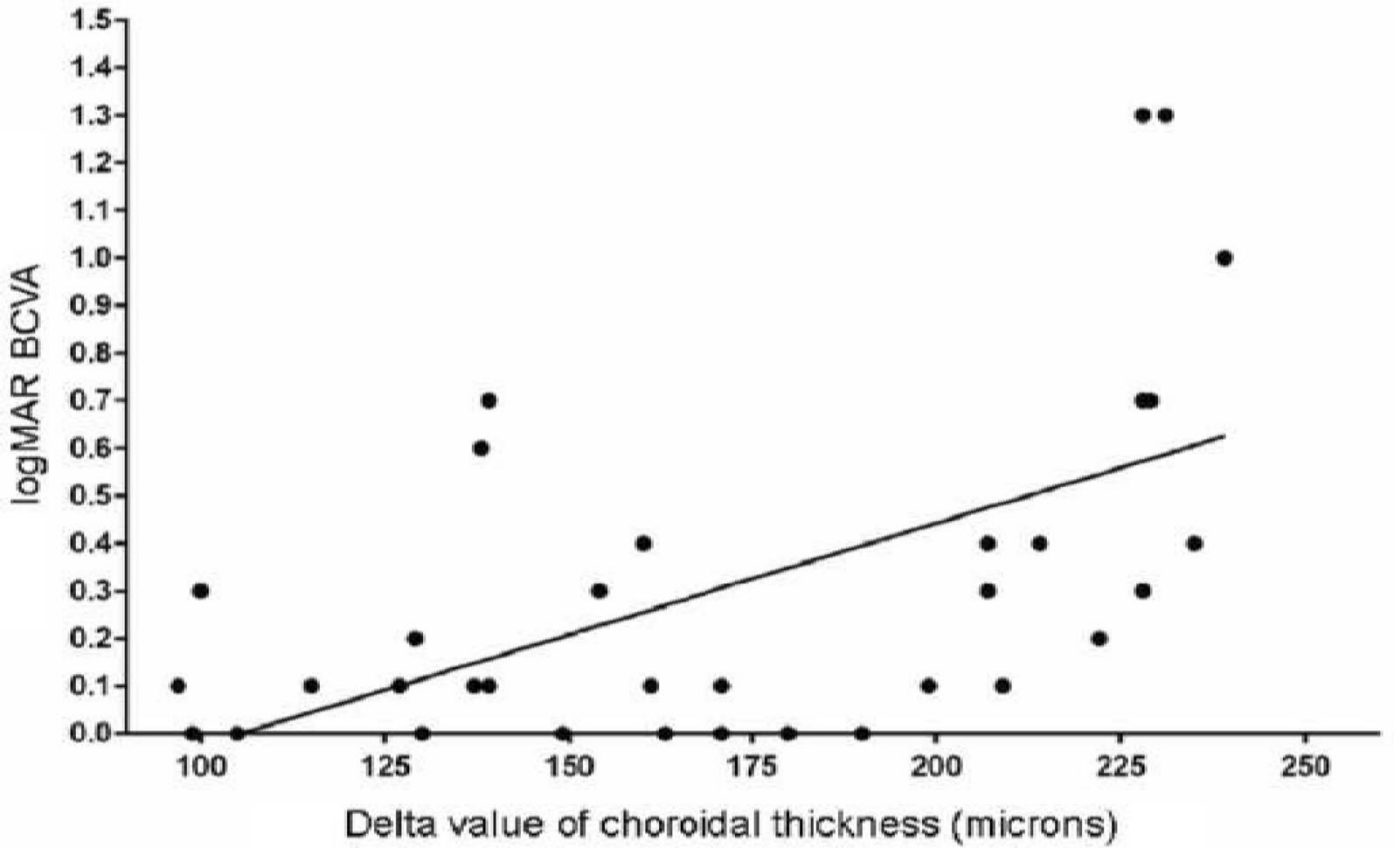
Correlation between delta value (difference between the mean choroidal thickness and actual value) of choroidal thickness and logMAR best corrected visual acuity (BCVA) in patients with staphyloma (37 eyes)

**Fig. 7:**
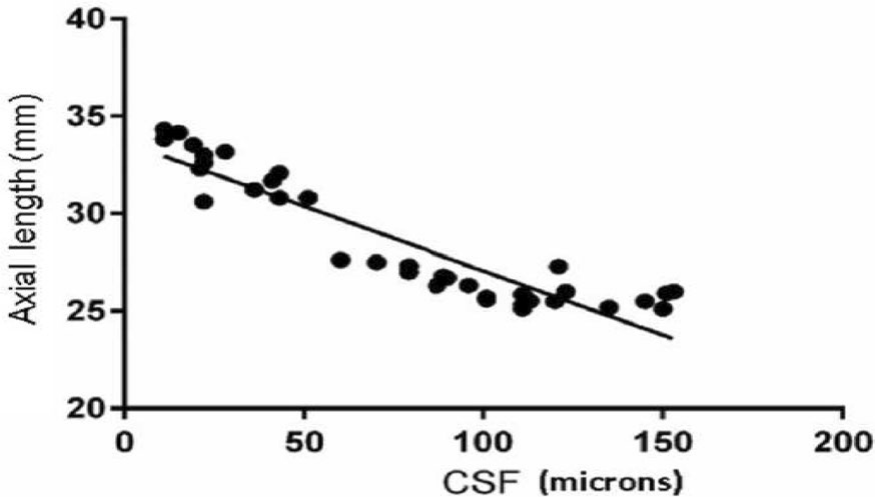
Correlation between central subfield (CSF) choroidal thickness and axial length in staphyloma subjects (37 eyes) (*r* = − 0.83, *r*_2_ = 0.67, *p* < 0.0001)

**Fig. 8:**
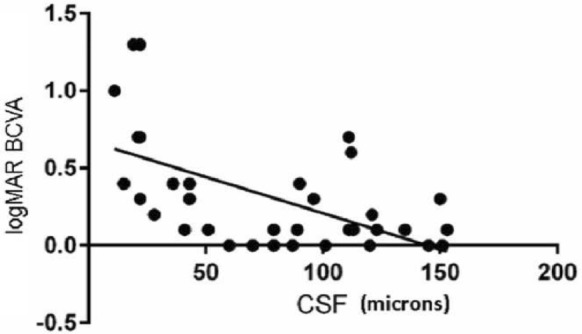
Correlation between central subfield choroidal thickness and logMAR best corrected visual acuity (BCVA) in staphyloma subjects (37 eyes), *r* = − 0.60; *r*_2_ = 0.35, *p* < 0.001

In staphyloma patients, CSF was 85.53 ± 48.61 μm. In the ETDRS subfields in staphyloma group, the outer nasal quadrant had the minimum thickness (57.32 ± 30.22 μm) and outer temporal quadrant had maximum thickness (96.83 ± 48.44 μm) (Fig. 2). In the normal cohort, with regard to race, the mean CSF was 251.88 ± 85.24 μm among white subjects, 253.85 ± 69.07 μm among Asian subjects, and 243.86 ± 48.30 μm among Black subjects (*p* = 0.87). There was a high statistically significant difference (*p* < 0.0001) on comparing choroidal thickness of myopia patients with that of normal subjects in all nine ETDRS subfields of choroidal thickness map. In stepwise multiple regression analysis in staphyloma group, the axial length was associated the most with choroidal thickness. Pearson correlation coefficient (*r*) was calculated to establish the relationship between the choroidal thickness and axial length (Fig. 3) in the entire cohort (123 eyes) which was statistically significant (*r* = − 0.71, *r*_2_ = 0.5, *p* < 0.0001). Figure 4 depicts that subfield choroidal thickness was inversely correlated with the logMAR BCVA (*r* = − 0.47; *r*_2_ = 0.22, *p* < 0.001) for entire cohort. Further, delta values were calculated from the mean values. Pearson correlation coefficient of delta value of choroidal thickness in staphyloma patients was statistically significant with axial length (Fig. 5) [*r* = − 0.75; *r*_2_ = 0.6, *p* < 0.001] and with logMAR BCVA (Fig. 6) [*r* = − 0.71; *r*_2_ = 0.5, *p* < 0.001]. Additionally, Pearson correlation coefficient was statistically significant between choroidal thickness and axial length (Fig. 7) [*r* = 0.083/*r*_2_ = 0.67] and logMAR BCVA (Fig. 8) [*r* = 0.6/*r*_2_ = 0.35] in eyes with staphyloma. Results were statistically significant but the correlation coefficient value was strong for axial length and moderate for logMAR BCVA comparison. There was a significant correlation between the refractive error and choroidal thickness in staphyloma group (*r* = 0.53/*r*_2_ = 0.3).

## Discussion

Choroidal blood flow is the highest in the body to satisfy the metabolic demands of the outer retina [7]. A very thin choroid as found in highly myopic eyes may deliver decreased amounts of oxygen and nutrients to the outer retina. This may affect signal generation by the photoreceptors or loss of overlying photoreceptors leading to decreased vision. OCT has emerged as an important imaging method in the evaluation and management of choroidal conditions. Ultrasonography (B-scan), though sometimes used to evaluate choroid, has limited clinical applicability due to poor resolution [8]. On the other hand, indocyanine green (ICG) angiography reveals useful clinical information about its vascularity but is invasive and does not provide cross-sectional images [9]. Spaide et al. [10] had proposed enhanced depth imaging (EDI) technique of imaging the RPE-Bruch’s choriocapillaris complex using Spectralis OCT which allows consistent choroidal visualization in most eyes. However, EDI system cannot create 3-dimensional choroid maps as the choroidoscleral boundary is not well delineated because of lack of deep penetration [11, 18].

Recently, there were several attempts to use SS-OCT to evaluate choroidal thickness and choroidal volume [12]. SS-OCT devices employ a longer light wavelength (1050 nm versus 840 nm with SD-OCT) that eliminates scattering of light and outline choroid-scleral border more distinctly. Pathological axial myopia is defined as a refractive error with a lower limit of − 6D along with associated posterior pathological changes and in absence of other (corneal, lenticular) causes of myopia [1]. It usually starts in early childhood (5–10 years of age) and progresses usually until the third decade [1]. The prevalence of high myopia increases with age and is 0.1% in preschool children, 0.5% in high school students and 1.5% in university students [13]. The proportion of patients with high myopia in the general population ranges from 1 to 2% in the United States, 5–8% in Japan, and 15% in Singapore [5]. The prevalence of posterior staphyloma increases from 1.4% of eyes with axial lengths of 26.5–27.4 mm to 71.4% of those measuring 33.5–36.6 mm and there was a significant correlation between posterior staphyloma and chorioretinal atrophy with 77.5% of staphylomatous eyes having degenerative changes [14]. In addition, degenerative changes were more apparent with increasing age and were invariably present in staphyloma eyes of individuals over 40 years [14].

In this present study, we compared the choroidal thickness in posterior scleral staphyloma patients with that of normal subjects using automated segmentation with SS-OCT. In several previous studies that used SD-OCT, CT was obtained manually for only the subfoveal region [4–6, 15, 16, 18]. The Topcon DRI-OCT, which was used in this study, enables automatic creation of choroidal thickness map. The average CSF thickness in patients with staphyloma was 85.53 ± 48.61 μm as compared to normal 250.24 ± 71.01 μm. Stepwise multiple regression analysis confirmed that choroidal thickness measured in staphyloma patients, correlated with axial length and visual acuity. The choroid was the thinnest nasally, both in staphyloma and normal group. There was a strong correlation between the axial length and choroidal thickness (*p* < 0.001). With increase in axial length there was corresponding decrease in choroidal thickness. In some patients with staphyloma, the choroidal thickness was even less than 50 μm. This correlates with the previously published histological reports of loss of RPE and choroid in patients with staphyloma [2]. We noted there was a moderate correlation between the BCVA and choroidal thickness in staphyloma group (Fig. 8; *r* = 0.6), but there was a strong correlation when axial length was compared with choroidal thickness (Fig. 7, *r* = 0.83). This explains that visual acuity may not be a true predictor of the severity of staphyloma. Two of the patients in staphyloma group had BCVA of 20/400 (logMAR BCVA 1.3) due to macular scar. This study also showed moderate correlation of refractive error with choroidal thickness (*r* = 0.5; *r*_2_ = 0.3). This can be probably be explained by the variation in degree of myopia in staphyloma group. However, there was a linear decrease in choroidal thickness with increase in refractive error. Sixteen of the 37 eyes in staphyloma group had a BCVA of 20/25 or better (Fig. 8). In these eyes, staphyloma was peripapillary and did not involve macula. This suggests that choroidal loss in macula from staphyloma could be the reason of compromised vision.

Ikuno et al. [17], using SD-OCT, reported that refractive error and posterior staphyloma height significantly correlated with choroidal thickness (CT) at the fovea but did not correlate with refractive status (like present study). Similarly, studies by Shao [5], Nashida et al. [6], and Zhou et al. [18] used SD-OCT for measuring CT showed comparable results to present study. Tan et al. [4] have reported the comparison of choroidal thickness measurement in normal subjects using SS-OCT and SD-OCT in nine ETDRS subfields. The measurements reported by them are comparable to our normal cohort.

The present study was different than the prior studies in several ways. SS- OCT was used to measure choroidal thickness, using automated analysis and thus better repeatability. The patients included in staphyloma group all had myopia of > 9D and axial length greater than 25 mm, higher than reported in the previous studies. Prior studies [4] did not take diurnal variation into consideration; however, in the present study, all OCT measurements were done between 8:00 AM and 10:00 AM. The staphyloma group in present study was compared to normative database avoiding the bias of having variable study population. The present study was a cross sectional study with a limited sample size (*n* = 37) and included participants in adult age group (median > 35 years in normal group and > 45 years in staphyloma group). Hence, the results of our study, may not be directly applicable to children and adolescents.

In conclusion the present study showed that choroidal thickness measured with SS-OCT strongly correlates with degree of myopia, visual acuity and positively correlates with axial length of the eye. Clinically, choroidal thickness measured with SS-OCT is a good predictor of visual acuity in this group of patients.

## Data Availability

Available.
